# Supplementary material for: Community Initiatives to Promote Basic Life Support Implementation—A Scoping Review
Source: J Clin Med. 2021 Dec 7;10(24):5719. doi: 10.3390/jcm10245719 (PMC8703423; doi:10.3390/jcm10245719)
Supplement: Supplementary file 1 [file jcm-10-05719-s001.zip › jcm-1492705-supplementary.pdf]

Supplementary Material 1: Search strategy applied

PubMed

- (((("Heart Arrest"[Mesh] OR "heart arrest*"[TIAB] OR "cardiac arrest*"[TIAB] OR "cardiovascular arrest*"[TIAB] OR "cardiopulmonary arrest*"[TIAB] OR "cardio-pulmonary arrest*"[TIAB] OR "Out-of-Hospital Cardiac Arrest*"[Mesh] OR OHCA OR "Out of Hospital Cardiac Arrest*"[TIAB] OR "out-of-hospital cardiac arrest*" [TIAB] OR "Outside-of-Hospital Cardiac Arrest"[TIAB]) OR (resuscitation [Mesh] OR resuscitation* [TIAB] OR "cardiopulmonary resuscitation"[Mesh] OR "cardiopulmonary resuscitation"[TIAB] OR "Cardio-Pulmonary Resuscitation" OR "Cardio Pulmonary Resuscitation" OR CPR [TIAB] OR "Life Support Care"[Mesh] OR "Basic Cardiac Life Support" OR "basic life support" OR "Cardiac Life Support" [TIAB] OR "cardiorespiratory resuscitation"[TIAB] OR "Heart Massage*"[Mesh] OR “heart massage*”[TIAB] OR “cardiac massage*” [TIAB] OR “chest compression*”[TIAB] OR “cardiac compression*”[TIAB]) OR (defibrillators [Mesh] OR defibrillator* [TIAB] OR “automated external defibrillator*” OR AED OR “External Defibrillator*” OR “Electric Shock Cardiac Stimulator*” OR “Electric Defibrillation” OR Electric Countershock [Mesh] OR “Electrical Cardioversion*” [TIAB] OR “Cardiac Electroversion*”))AND (bystander*[TIAB] OR "first responder*"[TIAB] OR "first-responder*"[TIAB] OR Layperson*[TIAB] OR “lay people”[TIAB] OR “lay rescuer*”[TIAB] OR “lay public” OR witness*[TIAB] OR “non-healthcare professional” [TIAB])) AND (((community OR public OR local OR social OR population* OR citizen*) AND (initiative* OR intervention* OR action* OR participation OR involvement* OR engagement OR preparation* OR implement* OR project* OR strategy* OR program OR programs OR network* OR training* OR campaign* OR education OR coaching OR information* OR learning OR instruction* OR guidance* OR response* OR responsiveness OR reply OR reaction OR awareness OR alertness OR realization OR sensibility OR sensitivity

OR consciousness) OR “community-based initiative*” OR “community-driven initiative*”))

- Search performed on 01/02/2021
- Filters: Only humans

EMBASE

- ((('heart arrest' OR 'cardiac arrest*' OR 'cardiovascular arrest*' OR 'cardiopulmonary arrest*' OR 'cardio-pulmonary arrest' OR 'out of hospital cardiac arrest' OR ohca OR 'out-of-hospital cardiac arrest*' OR 'outside-of-hospital cardiac arrest') OR ('heart massage OR 'cardiopulmonary resuscitation' OR 'cardio-pulmonary resuscitation' OR 'cardio pulmonary resuscitation' OR cpr OR 'basic life support' OR 'cardiorespiratory resuscitation' OR 'heart massage*' OR 'cardiac massage*' OR 'chest compression*' OR 'cardiac compression*' OR defibrillator* OR “automated external defibrillator*” OR AED OR “External Defibrillator*” OR “Electric Shock Cardiac Stimulator*” OR “Electric Defibrillation” OR Electric Countershock OR “Electrical Cardioversion*” OR “Cardiac Electroversion*”)) AND ('layperson' OR bystander* OR 'first responder*' OR 'first-responder*' OR layperson* OR 'lay people' OR 'lay rescuer*' OR 'lay public' OR witness* OR 'non-healthcare professional')) AND ((community OR public OR population* OR citizen*) AND (initiative* OR intervention* OR action* OR participation OR involvement* OR engagement OR implement* OR program OR programs OR network* OR training* OR campaign* OR guidance* OR response* OR responsiveness OR reply OR awareness OR alertness OR sensibility OR sensitivity OR consciousness OR 'community-based initiative*' OR 'community-driven initiative*’)))
- Search performed on 01/02/2021
- No filters

COCHRANE

- (MeSH descriptor: [Heart Arrest] OR ("cardiac arrest" OR "cardiovascular arrest*" OR "cardiopulmonary arrest*" OR "cardio-pulmonary arrest*"):ti,ab,kw OR MeSH descriptor: [Out-of-Hospital Cardiac Arrest] OR ("cardiopulmonary resuscitation" OR "Cardio Pulmonary Resuscitation" OR CPR OR "Life Support Care" OR "Basic Cardiac Life Support" OR "basic life support" OR "Cardiac Life Support" OR "cardiorespiratory resuscitation"):ti,ab,kw OR MeSH descriptor: [Heart Massage] OR ("cardiac massage*" OR "chest compression*" OR "cardiac compression"):ti,ab,kw OR defibrillator* OR “automated external defibrillator*” OR AED OR “External Defibrillator*” OR “Electric Shock Cardiac Stimulator*” OR “Electric Defibrillation” OR Electric Countershock OR “Electrical Cardioversion*” OR “Cardiac Electroversion*”):ti,ab,kw) AND ((bystander* OR "first responder*" OR "first-responder*" OR Layperson* OR “lay people” OR “lay rescuer*” OR “lay public” OR witness* OR “non-healthcare professional”):ti,ab,kw) AND (community OR public OR local OR social OR population* OR citizen* OR person OR people):ti,ab,kw AND (initiative* OR intervention* OR action* OR participation OR involvement* OR engagement OR preparation* OR implement* OR project* OR strategy* OR program OR programs OR network* OR training* OR campaign* OR education OR coaching OR information* OR learning OR instruction* OR guidance* OR response* OR responsiveness OR reply OR reaction OR awareness OR alertness OR realization OR sensibility OR sensitivity OR consciousness OR “community-based initiative*” OR “community-driven initiative*”):ti,ab,kw
- Search performed on 01/02/2021
- No filters
